# Midwifery workforce profile in Limpopo Province referral hospitals

**DOI:** 10.4102/phcfm.v6i1.573

**Published:** 2014-04-25

**Authors:** Sam T. Ntuli, Gboyega A. Ogunbanjo

**Affiliations:** 1 Department of Public Health Medicine, University of Limpopo, Polokwane Campus, South Africa; 2 Department of Family and Primary Health Care, University of Limpopo, Medunsa Campus, South Africa

## Abstract

**Background:**

In sub-Saharan Africa including South Africa, maternal mortality rates remain unacceptably high due to a shortage of registered nurses with advanced midwifery diplomas.

**Objective:**

To determine the profile of registered nurses (RNs) involved in maternity care in public referral hospitals of the Limpopo Province, South Africa.

**Method:**

A cross-sectional descriptive study was conducted in all maternity units of Limpopo's public referral hospitals. The study population comprised of 210 registered nurses, who became the study sample. Data on their educational profile and work experience in midwifery was analysed using STATA version 9.0.

**Results:**

The mean age of the 210 registered nurses was 44.5 ± 9.1 years (range 21 to 62). The majority (152/210; 70%) were 40 years and older, 56% (117/210) had been working for more than 10 years, and 63/210 (30%) were due to retire within 10 years. Only 22% (46/210) had advanced midwifery diplomas, i.e. after their basic undergraduate training. Only six (2.9%) of the RNs providing maternity care in these referral hospitals were studying for advanced midwifery diplomas at the time of the study.

**Conclusion:**

This study demonstrated a shortage of registered nurses with advanced midwifery training/diplomas in referral hospitals of the Limpopo Province. This has a potentially negative effect in reducing the high maternal mortality rate in the province.

## Abstrait

**Le profil de la pratique de sage-femme dans les hôpitaux de recours**.

**Présentation:**

En Afrique subsaharienne, Afrique du Sud comprise, les taux de mortalité maternelle reste à des niveaux élevés inacceptables en raison d'un manque d'infirmières qualifiées disposant également d'un diplôme de sage-femme.

**Objectif:**

Déterminer le profil des infirmières qualifiées (IQ) impliquées dans les soins de santé maternelle dans les hôpitaux de recours publics de la province du Limpopo, en Afrique du Sud.

**Méthode:**

Une étude descriptive transversale a été réalisée dans tous les services de maternité des hôpitaux de recours publics de la province du Limpopo. La population de l’étude était composée de 210 infirmières qualifiées (IQ), qui ont constitué l’échantillon de l’étude. Les données relatives à leur profil éducatif et à leur expérience professionnelle dans la profession de sage-femme ont été analysées à l'aide du logiciel STATA version 9.0.

**Résultat:**

Les 210 infirmières qualifiées étaient en moyenne âgées de 44,5 ± 9,1 ans (fourchette allant de 21 à 62 ans). La majorité d'entre elles (152/210; 70%) était âgée de 40 ans et plus, 56% (117/210) d'entre elles exerçaient depuis plus de 10 ans, et 63/210 (30%) devaient prendre leur retraite dans les 10 années suivantes. Seules 22% (46/210) d'entre elles étaient titulaires de diplômes de sage-femme d'un niveau supérieur à leur formation de premier cycle de base. Seulement six (2.9%) IQ fournissant des soins de santé maternelle dans ces hôpitaux de recours suivaient des études pour obtenir leur diplôme de sage-femme au moment de l’étude.

**Conclusion:**

Cette étude a mis en avant un manque d'infirmières qualifiées disposant également d'une formation/d'un diplôme de sage-femme dans les hôpitaux de recours de la province du Limpopo. Ceci a un impact potentiellement négatif sur la réduction du fort taux de mortalité maternelle dans la province.

## Introduction

The World Health Organization (WHO) reported a global reduction of maternal mortality ratio (MMR) from 400 to 210 per 100 000 live births between 1990 and 2010.^1^ Relatively acceptable MMR is between 12 and 15 deaths per 100,000 live births based on available USA data obtained between 2003 and 2007.^1^ Progress is being made in both developed and developing countries, however, MMR remains unacceptably high in sub-Saharan Africa, despite average reductions from 850 to 500 per 100 000 live births.^2^ The global decrease in MMR has been attributed to an increase in the proportion of deliveries attended to by skilled health personnel.^2^ In South Africa (SA), the National Committee for the Confidential Enquiries into Maternal Deaths (NCCEMD) reported a 22% increase in MMR between 2005/2007 and 2008/2010, with the leading causes of death remaining unchanged, namely non-pregnancy related infections (HIV/AIDS), obstetric haemorrhage and hypertension.^3^ This implies that SA may probably miss its Millennium Development Goal (MDG) 5 target of reducing its MMR by 75% by 2015. The Limpopo province recorded a district MMR of 275.9 per 100 000 live births (Capricorn district) and 616 maternal deaths (12% of the country's total maternal deaths) between 2008 and 2010.^3^ In South Africa, a total number of 4867 maternal deaths was reported between 2008 and 2010.^3^

In South Africa, nursing education and practice have undergone changes in response to the demands of current clinical practice. Amongst the strategies used by SA to reduce MMR in meeting its MDG5 target, has been to improve the knowledge and skills of healthcare workers involved in maternal care.^3^ The Department of Health (DoH) initiated a programme of diploma and master's degree to train registered nurses known as ‘clinical nurse specialists’ (advanced midwives) who will be able to function with the increased responsibility of working independently in maternity units. However, little information is available on the proportion of RNs with advanced midwifery training involved in maternal care. In order to assist local healthcare teams to plan the training requirements for an advanced midwifery workforce, there is a need to identify the proportion of registered nurses with advanced midwifery training who provide maternity services in their settings.

A cross-sectional, descriptive study was undertaken to establish the profile of registered nurses (RNs) involved in maternity care in referral hospitals of the Limpopo Province, South Africa. These referral facilities were chosen because health care providers at lower levels of the health system, who may lack midwifery skills, seek the assistance of health care providers at higher levels of care. These health care providers are supposed to be better equipped or specially trained to provide guidance in the management, or to take over the responsibility of care for a particular episode of a patient's clinical condition.

## Method

The study was carried out in all maternity units of the referral hospitals in Limpopo Province, South Africa. A questionnaire which was initially piloted to ensure validity and reliability was distributed to all referral hospitals comprising of (4) four regional hospitals and (1) one tertiary hospital complex (comprising of two hospitals situated 30 km apart). Data was collected from either the maternity nursing manager, sister in charge of the maternity unit or the nursing manager of the various referral hospitals who signed consent forms before providing the required information on their nursing staff. Data reflected the demographic and educational profile of registered nurses practicing midwifery in March 2013. The statistical software, STATA version 9.0 was used for data analysis.

## Ethical considerations

Ethics approval was obtained from the Polokwane/Mankweng hospital complex Research Ethics Committee (PMREC) of the University of Limpopo (Polokwane Campus) in South Africa (Ethics clearance certificate number: PMREC 50/2013). Permission to obtain the demographic and educational data of the nurses was obtained from the Limpopo Province Research Committee. Anonymity and confidentiality of data was assured by group data analysis without any personal identifiers.

## Results

**FIGURE 1:**
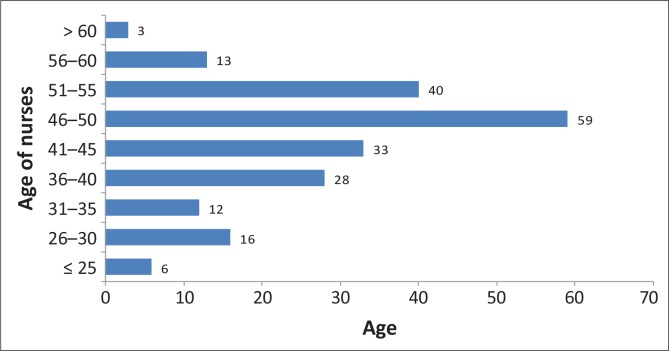
Age differences for registered nurses.

There were 210 registered nurses working in the various maternity units of the referral hospitals in the Limpopo Province during March 2013. Figure 1 shows the age distribution of the RNs who provided maternal care in these referral hospitals during the study period. The mean age of these nurses was 44.5 ± 9.1 years (range 21 to 62 years). The majority (152/210; 70%) were 40 years and older.

**FIGURE 2:**
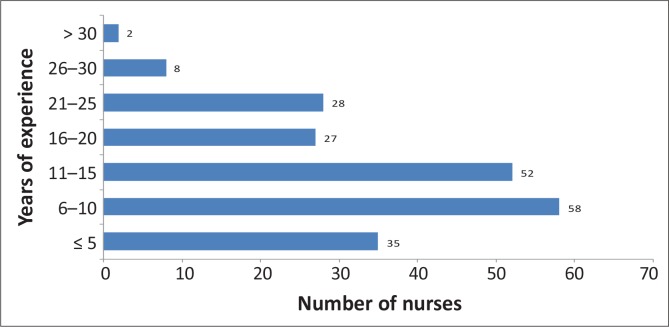
Years of experience of registered nurses (RNs) working in maternity facility.

Analysis of work experience is presented in Figure 2, and shows that 56% (117/210) had been working for more than 10 years as registered nurses involved in maternity care. Almost 14% (30/210) had less than 5 years’ work experience. Of these (*n* = 30), 60% (18/30) were younger than 30 years of age.

Table 1 shows a brief profile of the postgraduate education levels achieved by the RNs involved in maternity care per health facility. Only 21.9% (46/210) of them had advanced midwifery diplomas obtained from various colleges of nursing or universities. Less than 3% (6/210) of the RNs based at the referral hospitals were studying for an advanced diploma in midwifery at the time of the study.

**TABLE 1:** Distribution of registered nurses with advanced midwifery training per facility.

Registered Nurses (RNs)	Tertiary Complex		Regional Hospitals			
	
	Hospital A	Hospital B	Hospital C	Hospital D	Hospital E	Hospital F
Number of RNs in maternity units	28	37	18	33	29	65
Number of RNs with advanced midwifery diplomas *N* (%)	6 (21%)	5 (14%)	8 (44%)	10 (30%)	6 (21%)	11 (17%)

Note: Fewer than 3% (6/210) of the RNs were studying for a postgraduate diploma in midwifery.

## Discussion

From our study, a small proportion, that is, approximately 22% (46/210) of the RNs had completed advanced midwifery training. This finding is in contrast to those studies conducted in a number of developed countries, which showed that more than 50% of midwives had completed postgraduate training in midwifery.^4, 5^ In addition, previous studies have shown that having a skilled attendant present at every maternal delivery reduces maternal deaths.^6, 7^ A recent global review suggests levels of maternal mortality in developing regions have fallen since 1990, but it has become increasingly clear that significant improvements in health care for women are required if the goal is to be achieved.^8^ This is the case for sub-Saharan Africa and South Asia, in which at least 87% of the estimated annual 342 900 maternal deaths worldwide occur according to recent estimates, with over 50% of all maternal deaths occurring in only six countries, namely India, Nigeria, Pakistan, Afghanistan, Ethiopia and the Democratic Republic of Congo. ^8^

From the questionnaire, the following were given as possible reasons for the high number of midwives without advanced midwifery training: shortage of staff due to high staff turnover and unavailability of training spaces at the nursing colleges. However, a study on job satisfaction amongst registered nurses (*n* = 34) in a community hospital in Limpopo Province, South Africa found that more than half (54%; 18/34) were dissatisfied with personal growth and development of staff.^9^ Another study reported that 69% (65/94) of registered nurses were absent from work because they had to do jobs that required more skills than they had.^10^

In addition to these challenges, the nursing population is also ageing. Our findings indicate that more than two-thirds of the registered nurses were 40 years and older, which means that half of the current group will retire within 10 to 15 years’ time. These findings concur with reports by Sipe and co-workers,^4^ as well as Bogossian and collaborators^5^, which reported that clustering occurred in the age range of 51–54 years and, that 73% (534/729) of practicing midwives were over 40 years of age respectively. It was interesting to note that 60% (126/210) of the registered nurses had been working in maternity wards for more than ten years, of whom only 22% had advanced midwifery diplomas. This indicates that registered nursing education and training in ‘maternal care’ in the Limpopo province occurs primarily through ‘on the job’ experience. Bogossian *et al*.^4^ indicated that the majority of midwives (61%; 305/729) in their study were working part-time, an average of 35 hours per week. In contrast, our study showed that all midwives worked full-time for an average of 40 hours per week. The implication of the latter is that the nurses working in these maternity units are relatively overworked and only slightly over a fifth had the advanced midwifery training to deal with the possible complications associated with maternal morbidity and mortality. Crowe *et al*.^11^ conservatively estimate that there will be between 130 and 180 million non-skilled birth attendant (SBA) births in South Asia and sub-Saharan Africa from 2011 to 2015 with 90% of these in rural areas. This means that there is an urgent need to step up advanced midwifery training of nurses if the province (with its high maternal mortality ratio) and South Africa are to achieve the MDG5 target by 2015.

### Limitations of the study

The findings of this study cannot be generalised as it did not extend to registered nurses providing maternal care in Primary Health Care (PHC) clinics/health centres and district hospitals. Nevertheless, the study demonstrates the level of training of RNs managing maternal cases in the maternity units of the referral hospitals where complicated or high risk maternal cases are sent from the primary care facilities.

## Conclusion

This study demonstrated a shortage of registered nurses with advanced midwifery training/diplomas in referral hospitals of the Limpopo Province. This has a potential negative effect in reducing the high maternal mortality rate in the province. The challenges raised in this study in terms of the profile of RNs managing these maternity units require urgent interventions to fast track advanced midwifery training programmes to upskill the RNs currently employed in the province. Additional research is needed to examine the profile of registered nurses providing maternal care in the Primary Health Care (PHC) and district hospitals, as this will give a comprehensive picture of the training needs in advanced midwifery for Limpopo province.
